# Supplementary material for: Nurhachius luei, a new istiodactylid pterosaur (Pterosauria, Pterodactyloidea) from the Early Cretaceous Jiufotang Formation of Chaoyang City, Liaoning Province (China) and comments on the Istiodactylidae
Source: PeerJ. 2019 Sep 19;7:e7688. doi: 10.7717/peerj.7688 (PMC6754973; doi:10.7717/peerj.7688)
Supplement: Supplemental Information 1 [file peerj-07-7688-s001.docx]

***Nurhachius luei***, **a new** **istiodactylid pterosaur (Pterosauria, Pterodactyloidea) from the Early Cretaceous Jiufotang Formation of Liaoning Province (China) and comments on the group**

Xuanyu Zhou ^1,2^, Rodrigo Vargas Pêgas^3^, Maria Eduarda de Castro Leal^4,5^, Niels Bonde^5^

1. Institute of Geology, Chinese Academy of Geological Sciences, Beijing, China

2. China University of Geosciences, Beijing, China

3. Laboratory of Vertebrate Paleontology and Animal Behavior, Universidade Federal do ABC, São Bernardo, São Paulo, Brazil

4. Departamento de Geologia, Universidade Federal do Ceará, Fortaleza, Ceará, Brazil

5. Zoological Museum (SNM), Copenhagen University, Copenhagen, Denmark

**SUPPLEMENTARY MATERIAL**

**Character list for the data matrix (per anatomical region)**

**CRANIUM**

1. External naris (or nasoantorbital fenestra), position relative to the premaxilla (Andres et al. 2014: character 48; modified from Kellner 2001: character 5):

0 - main part dorsal to the ventral margin of the premaxilla

1 - main part displaced posterior to the premaxilla

1. External naris, dorsoventrally compressed:

0 - absent

1 – present

1. External naris and antorbital fenestra, configuration (rephrased from Unwin 1995):

0 - separated

1 - confluent, forming a nasoantorbital fenestra

1. External naris and antorbital fenestra (or nasoantorbital fenestra), ventral margin length relative the skull length (rephrased from Kellner 2001: character 7):

0 - shorter than 40% of the skull length

1 - longer than 40% of the skull length

1. Antorbital (or nasoantorbital) fenestra, posterior margin, shape (modified from Unwin 1995):

0 - straight

1 - concave

1. Nasoantorbital (or antorbital) fenestra extending dorsal to the orbit:

0 - absent

1 - present

1. Orbit, shape (modified from Kellner 2004: character 10; combined with Andres & Ji 2008: character 9):

0 - subcircular

1 - quadrangular (broad base)

2 - piriform (dorsoventrally elongated)

1. Orbit, comparatively small:

0 - absent

1 - present

1. Ventral margin of the orbit:

0 - closed

1 - open

1. Orbit, position (Andres & Myers 2013: character 45; modified from Kellner & Langston 1996; Kellner 2001: character 9; and Andres & Ji 2008: character 10):

0 - middle of the skull, with the ventral margin of the orbit below the middle of the antorbital (or nasoantorbital) fenestra and the dorsal margin of the orbit above the dorsal margin of the antorbital (or nasoantorbital) fenestra

1 - high in the skull, with the dorsal margin of the orbit surpassing the level of the nasoantorbital fenestra

2 - low in the skull, with the entire orbit lower than the dorsal margin of the antorbital (or nasoantorbital) fenestra

1. Suborbital opening (Kellner 2001: character 10):

0 - absent

1 - present

1. Lower temporal fenestra, shape (Wang et al. 2012: character 15):

0 - comparatively broad, with extensive subhorizontal ventral margin (trapezoidal)

1 - piriform, with dorsal portion wider than ventral

2- piriform, with ventral portion wider than dorsal

3 - reduced (slit-like)

1. Lower temporal fenestra, position relative to orbit (Andres et al. 2014: character 57):

0 - posterior to orbit

1 - reaches under posterior margin of orbit

1. Choanae, separation:

0 - separated by vomer

1 - confluent

1. Postpalatine fenestra, shape:

0 - quadrangular/subriangular

1 - oval

2 - egg-shaped

3 - elongated egg-shaped

4 - kite-shaped, rounded margins

1. - elliptical
2. - reduced, slit-like
3. Secondary subtemporal fenestra:

0 - absent

1 - present

1. Interpterygoid fenestra, size (modified from Kellner 2001: character 26):

0 - smaller than subtemporal fenestra

1 - larger than subtemporal fenestra

2 - extremely reduced

1. Interpterygoid fenestra, shape:

0 - compressed laterally

1 - broad, longer than wide

2 - compressed anteroposteriorly, wider than long

3 – round

1. Pterygoid fenestra:

0 - absent

1 - present

1. Upper and lower jaw, marked gap during occlusion:

0 - absent

1 - present

1. Upper and lower jaw, shape:

0 – compressed

1 – broad

1. Skull, main part of dorsal margin, curvature excluding cranial crest (modified from Kellner 2001: character 3):

0 - nearly straight

1 - concave
 2 – convex

1. Skull, height, exclusive of cranial crests (modified from Witton, 2012)

0 – under 25% of total jaw length

1 – over 25% of total jaw length

1. Jaws, anterior taper (modified from Andres *et al.*, 2014)

0 – attenuated

1 – subparallel

1. Length of the rostrum (pm-naof) relative to the skull length (pm-sq) (modified from Kellner 2001: character 1):

0 - reduced

1 - elongated (about or less than half of skull length)

2 - extremely elongated (more than half of skull length)

1. Rostral end (premaxilla), shape:

0 - rounded

1 - pointed

2 - sharp tip

3 - flat surface

1. Rostral end of premaxillae/maxillae downturned:
    0 - absent
    1 - present
2. Rostrum, distinct concavity on occlusal surface:

0 - absent

1 - present

1. Rostrum, anterior portion forming a high ossified plate:

0 - absent

1 - present

1. Premaxillae, anterior horizontal expansion (modified from Kellner 2001: character 12):

0 - absent
 1 - present, with premaxillary end high
 2 - present, with premaxillary end dorsoventrally flattened

1. Premaxillae anterior expansion, shape in horizontal plane (Andres & Myers 2013: character 37; modified from Kellner 2001: character 12):

0 - elliptical

1 - anteriorly expanded

2 - quadrangular

3 - absent

1. Premaxillae, anterior end rodlike:

0 - absent

1 – present

1. Premaxillary process separating the external nares, thickness (Kellner 2001: character 2):

0 - wide

1 – narrow

1. Premaxilla, posterodorsal margin of nasoantorbital fenestra (including nasal), width (modified from Andres et al. 2014: character 58; rephrased from Lü et al. 2009: character 8):

0 - wide

1 - thin

1. Premaxillary sagittal crest (modified from Kellner 2003: character 12):

0 - absent

1 - present

1. Premaxillary sagittal crest, position (modified from Kellner 2003: character 12):

0 - confined to the anterior portion of the skull
 1 - starting anterior to the anterior margin of the nasoantorbital fenestra, extending beyond occipital region
 2 - starting at about the anterior margin of the nasoantorbital fenestra, reaching the skull roof above the orbit but not extending over the occipital region
 3 - starting close or at the anterior portion of the skull and extended over the occipital region
 4 - starting at the posterior half of the nasoantorbital fenestra.
 5 - starting at the middle portion of the nasoantorbital fenestra, extending above the occipital region

1. Premaxillary sagittal crest, shape (Andres & Myers 2013: character 52):

0 - striated, low with a nearly straight dorsal margin
 1 - striated, high with a nearly straight dorsal margin

2 - round dorsal margin, blade-shaped
 3 - smooth, expanded anteriorly and forming a low rod-like extension posteriorly

4 - smooth, starting low anteriorly and very expanded posteriorly

5 - striated, low with convex dorsal margin

1. Premaxillary crest, elongated dorsal premaxillary spike-like projection (rephrased from Martill & Naish 2006: character 2):

0 - absent

1 - present

1. Premaxillary crest, distinct expansion on the anterior part (Pinheiro et al. 2011: character 9):

0 - absent

1 - present

1. Premaxillary crest, concentric striae on the anterior region (new character):

0 - absent

1 - present

1. Premaxillary crest, anterior margin, strong reentrancy at the base (new character):

0 - absent

1 – present

1. Premaxilla, posterior dorsal process, curved upward:

0 - absent

1 - present

1. Maxilla, posterior ventral expansion (Kellner 2001: character 13):

0 - absent

1 – present

1. Maxilla-nasal contact (modified after Unwin 2003: character 13):

0 - absent

1 - present

1. Maxilla-nasal contact, broadness (Unwin 2003: character 13):

0 - broad

1 - narrow

1. Nasal descending process, position (modified after Kellner 2001: character 14):

0 - absent

1 - present

1. Nasal descending process, position (modified after Kellner 2001: character 14):

0 - placed laterally

1 - placed medially

1. Nasal descending process, length (modified after Kellner 2003: character 15, Andres & Ji 2008: character 23):

0 - long, almost reaching the ventral margin of the skull

1 - short

2 - knob-like (extremely reduced)

1. Nasal descending process, orientation (modified after Kellner 2003: character 15):

0 - inclined anteriorly

1 - subvertical

1. Nasal descending process, lateral foramen (rephrased from Kellner 2003: character 16):

0 - absent

1 – present

1. Lacrimal, extensive fenestration:

0 - absent

1 - present

1. Lacrimal, orbital process (modified from Andres & Myers 2013: character 66):

0 - absent

1 - present

1. Jugal, lacrimal process base, width (modified from Kellner 2004: character 18):

0 - broad

1 – narrow

1. Jugal, lacrimal process, inclination (modified from Kellner 2004: character 18):

0 - inclined anteriorly

1 - subvertical

2 - inclined posteriorly

1. Jugal, presence of pronounced ridge on the lateral side:

0 - absent

1 - present

1. Jugal, postorbital process, orbital process (Andres et al. 2014: character 98):

0 - absent

1 - present

1. Quadrate, inclination relative to ventral margin of skull (based on Unwin 1995; rephrased from Kellner 2001: character 18):

0 - anteriorly

1 - subvertical

2 - inclined about 120° posteriorly

3 - inclined about 150° posteriorly

1. Cranio-mandibular articulation, position relative to orbit (modified from Kellner 2001: character 19):

0 - posterior to posterior margin of orbit

1 - under center of orbit

2 - under anterior margin of the orbit

3 - anterior to the anterior margin of orbit

1. Helical jaw joint (Bennett 1994: character 5):

0 - absent

1 - present

1. Frontal, anterior portion rugose (Wang et al. 2005):

0 - absent

1 - present

1. Frontal, ossified crest (Bennett 1994: character 30):

0 - absent

1 - present

1. Frontal, ossified crest, position (Wang et al. 2012):

0 - confined to posterior end of skull
 1 - starting above orbit
 2 - starting on posterior half of nasoantorbital fenestra

1. Frontal, ossified crest, shape (modified from Kellner 2001: character 15)

0 - low, blunt
 1 - short, spike-like, dorsally deflected

2 - spike-like, directed posteriorly

3 - narrow, broad, directed posteriorly
 4 - low, broad base, fan-shaped

5 - high, broad base, crown-shaped

6 - high, broad base, casqued-shaped

7 - high, broad base, directed posteriorly, at least doubling height of skull above orbit.

1. Parietal, ossified crest (modified from Kellner 2001: character 16):

0 - absent
 1 - present

1. Parietal, ossified crest, shape (modified from Kellner 2001: character 16):

0 - blunt
 1 - constituting the base of the posterior portion of the cranial crest

2 - expanded, with rounded margin

1. Posterior region of the skull rounded with the squamosal displaced ventrally:
    0 - absent
    1 - present
2. Supraoccipital (Kellner 2001: character 20):

0 - does not extend backwards

1 - extends backwards

1. Supraoccipital, foramen (rephrased from Kellner 2001: character 21):

0 - absent

1 - present

1. Paroccipital processes, expanded distal ends (rephrased from Unwin 1995):

0 - absent

1 - present

1. Foraminae piercing the anterior portion of the palate, numerous:

0 - absent

1 - present

1. Palatal occlusal surface (modified from Kellner 2003: character 24):

0 - smooth

1 - discrete palatal ridge, tapering anteriorly
 2 - strong palatal ridge, tapering anteriorly
 3 - strong palatal ridge, confined to the posterior portion of the palate

1. Palate, slight expansion close to the anterior margin of the nasoantorbital (or naris + antorbital) fenestra:

0 - absent

1 - present

1. Palate, anterior tip, dorsal deflection (modified from Andres & Ji 2008; Rodrigues & Kellner 2013):

0 - absent

1 - present, slight; with the first pair of upper alveoli not completely surpassing the second pair

2 - present, strong; with the first pair of upper alveoli above the second pair

1. Maxilla and internal naris, (rephrased from Kellner 2001: character 25):

0 - contact

1 - do not contact

1. Palatines, shape:

0 - broad

1 - thin bars

1. Basisphenoid body, length (modified from Kellner 2001: character 23):

0 - shorter than wide

1 - longer than wide

**MANDIBLE**

1. Mandibular rostral end, opposing dentaries (rephrased from Unwin 1995; Kellner 2001: character 27)

0 - unfused

1 - fused

1. Mandibular rostral end, extension of the contact surface of opposing dentaries (Kellner 2001: character 27; Pêgas et al. 2016: character 52):

0 - short, limited to the tip

1 - short, extended posteriorly less than 30% of mandible length

2 - long, up to 55% the mandible length

3 - long, extended over 55 % of mandibular length

1. Mandibular rostral end, shape:

0 - rounded

1 - pointed

2 - sharp tip

1. Dentary, dorsal margin, distinct posterior eminence close to the separation of mandibular rami:

0 - absent

1 - present

1. Dentary, tip projected anteriorly:

0 - absent

1 - present

1. Dentary, tip, odontoid process

0 - absent

1 - present

1. Dentary ossified sagittal crest (modified from Kellner 2003: character 33):

0 - absent

1 - present

1. Dentary ossified sagittal crest, position (modified from Kellner 2003: character 33):

0 - confined to the anterior third of the lower jaw

1 - extending close to the middle portion of the jaw

1. Dentary ossified sagittal crest, shape (modified from Kellner 2001: character 30):

0 - shallow
 1 - blade-like
 2 - deep, broad in lateral view
 3 - elongated ridge

1. Dentary, posteroventral fossa: dentary fossa

0 - absent

1 - present

**DENTITION**

1. Teeth, position and presence:
    0 - present, evenly distributed along the jaws
    1 - absent from the anterior portion of the jaws
    2 - confined to the anterior part of the jaws
    3 - jaws toothless
2. Maxillary teeth, largest positioned posteriorly (rephrased from Unwin 1995: character X):

0 - absent

1 – present

1. Teeth, shape variation:

0 - isodont

1 - heterodont

1. Teeth, anterior, marked variation in size:

0 - absent

1 - present

1. Teeth, upper jaw, variation in the size of the anterior teeth with the 4th larger than the 5th and 6th (modified from Kellner 2001: character 33):

0 - absent

1 - present

1. Teeth, base broad and oval (rephrased from Unwin 1995):

0 - absent

1 - present

1. Teeth, serrated:

0 - present

1 - absent

1. Teeth, peg-like (cone-shaped) (Kellner 2001: character 35):

0 - absent

1 - present, 15 or less on each side of the jaws

2 - present, more than 15 on each side of the jaws

1. Teeth, small needle-shaped:

0 - absent

1 - present

1. Teeth, lateral compression:

0 - absent

1 - present, moderate

2 - present, strong

1. Teeth, sharp carinae:

0 - absent

1 - present

1. Teeth, anterior positions, relative elongation (modified from Kellner 2003: character 40):

0 – under twice as wide

1 – over twice as wide, under four times as wide

2 – over four times as wide

1. Teeth, fluted surface (modified from Andres et al. 2013: character 110):

0 - absent

1 - present

1. Teeth, crowns, with labial and lingual depressions

0 - absent

1 - present

1. Teeth, labial/lingual view, mesiodistal constriction between crown and root

0 - absent

1 - present

1. Teeth, labial cingulum

0 – absent

1 - present

1. Alveoli, lateral platform:

0 - absent

1 – present

1. Toothline, curvature between alveoli 4 and 8

0 – absent

1 - present

**AXIAL SKELETON**

1. Atlas and axis:

0 - unfused

1 – fused

1. Cervical vertebrae, postexapophyses:

0 - absent

1 - present

1. Mid-cervical vertebrae, centrum, lateral foramen:

0 - absent

1 - present

1. Mid-cervical vertebrae, length:
    0 - short, sub-equal in length

1 - longer than wide, with length less than 3 times width

2 - elongated, with length more than 3 times width

3 - extremely elongated

1. Mid-cervical vertebrae, ribs:

0 - present

1 - absent

1. Mid-cervical vertebrae, neural spines, height (Andres & Ji 2008: char. 67):

0 - tall

1 - low

2 - extremely reduced

1. Mid-cervical vertebrae, neural spines, shape (modified from Kellner 2001: character 43; Andres & Ji 2008: char 66):

0 - blade-shaped

1 - spike-shaped

2 - ridge

1. Dorsal vertebrae, fused into a notarium:

0 - absent

1 - present

1. Caudal vertebrae, quantity:

0 - more than 15

1 - 15 or less

1. Caudal vertebrae, zygapophyses forming rod-like ossified processes:

0 - absent

1 - present

1. Proximal caudal vertebrae centrum, centrum shape (rephrased from Bennett 1994: character 31):

0 - single

1 - duplex

**PECTORAL GIRDLE**

1. Scapula, length relative to coracoid length:
    0 - subequal or longer than coracoid
    1 - scapula shorter than coracoid (1 > sca/cor > 0.80)
    2 - substantially shorter than coracoid (sca/cor < 0.80)
2. Scapula, proximal end (rephrased from Kellner 2003: character 50):

0 - elongated

1 - sub-oval

1. Scapula, shape (rephrased from Kellner 2003: character 51):

0 - elongated

1 - stout, with constructed shaft

1. Coracoid, proximal end, shape:

0 - flattened

1 - oval

1. Coracoid, sternal articulation (modified from Kellner 2003: character 52):

0 - no developed articulation surface

1 - articulation straight or slightly concave

2 - articulation strongly concave

1. Coracoid, sternal articulation, posterior expansion:

0 - absent

1 - present

1. Coracoid, ventral margin, deep flange

0 - absent
 1 - present

1. Coracoid, broad tubercle on ventroposterior margin (Kellner 2004: character 57):

0 - absent

1 - present

1. Cristospine, shape (rephrased from Bennett 1994: character 34):

0 - absent

1 - shallow and elongated

2 - deep and short

**FORELIMB**

1. Humerus, proportional length relative to the metacarpal IV (hu/mcIV) (Kellner 2001: character 46):

0 - hu/mcIV > 2.50

1 - 1.50 < hu/mcIV < 2.50

2 - 0.40 < hu/mcIV < 1.50

3 - hu/mcIV < 0.40

1. Humerus, proportional length relative to the femur (hu/fe) (modified from Kellner 2001: character 47):

0 - hu/fe ≤0.80

1 - 1.4 > hu/fe > 0.80

2 - hu/fe > 1.40

1. Humerus plus ulna, proportional lengths relative to the femur plus tibia (hu+ul/fe+ti) (Kellner 2001: character 48):

0 - humerus plus ulna about 0.80% or less of femur plus tibia length

(hu+ul/fe+ti < 0.80)

1 - humerus plus ulna larger than 0.80% of femur plus tibia length

(hu+ul/fe+ti > 0.80)

1. Humerus, proximal end, foramen on dorsal surface near medial margin:

0 - absent

1 - present

1. Humerus, proximal end, foramen on ventral surface close to proximal margin (modified from Kellner 2001: character 49):

0 - absent

1 - present

1. Humerus, deltopectoral crest, shape (modified from Kellner 2003: character 58):

0 - reduced, positioned close to the humerus shaft

1 - enlarged, proximally placed, with almost straight proximal margin

2 - enlarged, hatchet shaped, proximally placed

3 - enlarged, hatched shaped, positioned further down the humerus shaft

4 - enlarged, warped

5 - long, proximally placed, curving ventrally

6 - enlarged, square outline

1. Humerus, medial (= ulnar) crest (modified from Kellner 2001: character 51):

0 - reduced

1 - directed posteriorly

2 - present, massive, with a developed proximal ridge

1. Humerus, distal articulation, shape (Andres & Myers 2013: character 159):

0 - oval or D-shaped
 1 - subtriangular

1. Humerus, between distal condyles, pneumatic foramen (Longrich et al. 2018: character 227):

0 - absent

1 - present

1. Ulna, proportional length relative to metacarpal IV (ul/mcIV) (Kellner 2003; modified from Kellner 2001: character 53):

0 - ulna 3.6 times longer than metacarpal IV (ul/mcIV > 3.6)

1 - length of ulna between 3.6 and two times the length of metacarpal IV (3.6 > ul/mcIV > 2)

2 - ulna between two times and the same length of metacarpal IV (2 > ul/mcIV > 1)

3 - ulna about the same length or smaller than metacarpal IV (ul/mcIV < 1)

1. Ulna and radius, diameter at midshaft (Kellner, 2003; modified from Bennett, 1994: character 33)

0 - subequal

1 - diameter of radius about half that of ulna

2 - diameter of radius less than half that of ulna

1. Distal syncarpals, shape (distal view) (modified from Kellner 2001: character 55):

0 - irregular

1 - from rectangular unit

2 - form triangular unit

1. Proximal syncarpal, shape (proximal view) (from Unwin, 2003)
   0 – quadrangular or irregular
   1 - pentagonal
2. Distal syncarpals, shape (distal view) (from Unwin, 2003)
   0 – irregular
   1 – rectangular unit
   2 – triangular unit
3. Pteroid:

0 - absent
 1 - shorter than half the length of the ulna
 2 - longer that half the length of the ulna

1. Pteroid, proximal articulation, expanded in right angle with the shaft:

0 - absent

1 - present

1. Metacarpals I - III, relation with carpus (modified from Bennett 1994: character 15):

0 - articulating with carpus

1 - metacarpal I articulates with carpus, metacarpals II and III reduced

2 - not articulating with carpus

1. Manual digit IV first phalanx, proportional length relative to metacarpal IV (ph1d4/mcIV) (modified from Kellner 2001: character 58):

0 - both small and reduced
 1 - both enlarged with ph1d4 over four times the length of mcIV (ph1d4/mcIV>4.0)

2 - both enlarged with ph1d4 between four and two times the length of mcIV (4.0>ph1d4/mcIV>2.0)

3 - both enlarged with ph1d4 about or less than two times the length of mcIV (2.0>ph1d4/mcIV>1.0)

4 - both enlarged with ph1d4 about the same or smaller than the length of mcIV (ph1d4/mcIV<1.0)

1. Manual digit IV first phalanx, proportional length relative to tibiotarsus (ph1d4/ti) (modified from Kellner 2001: character 59):

0 - ph1d4 reduced

1 - ph1d4 elongated and less than twice the length of ti (ph1d4/ti <2.00)

2 - ph1d4 elongated about or longer than twice the length of ti (ph1d4/ti >2.00)

1. Manual digit IV second phalanx, proportional length relative to first phalanx (ph2d4/ph1d4) (modified from Kellner 2001: character 60):

0 - both short or absent

1 - elongated with second phalanx about the same size or longer than first (ph2d4/ph1d4 larger than 1.00)

2 - elongated with second phalanx up to 30% shorter than first (ph2d4/ph1d4 between 0.70 - 1.00)

3 - elongated with second phalanx more than 30% shorter than first (ph2d4/ph1d4 smaller than 0.70)

1. Manual digit IV third phalanx, proportional length relative to first phalanx (ph3d4/ph1d4) (Kellner 2001: character 61):

0 - both short or absent

1 - ph3d4 about the same length or larger than ph1d4

2 - ph3d4 shorter than ph1d4

1. Manual digit IV third phalanx, proportional length relative to the second phalanx (ph3d4/ph2d4) (Kellner 2001: character 62):

0 - both short or absent

1 - ph3d4 about the same size or longer than ph2d4

2 - ph3d4 shorter than ph2d4

1. Manual digit IV forth phalanx, proportional length relative to the first phalanx of manual digit IV (ph4d4/ph1d4):

0 - both short or absent

1 - both elongated, with the forth phalanx longer than the first (ph4/d4/ph1d4>1.00)

2 - both elongated with the forth phalanx the same length or shorter, but longer than 35% the length of the first (1.00>ph4d4/ph1d4>0.35)

3 - both elongated with the forth phalanx less than 35% the length of the first (ph4d4/ph1d4<0.35)

**HIND LIMB**

1. Femur, length relative to metacarpal IV length (fe/mcIV) (modified after Kellner 2001, character 63) (Andres & Ji 2008: char 110):

0 - femur about twice or longer than metacarpal IV
 (fe/mcIV > 2.00)
 1 - femur longer but less than twice the length of
 metacarpal IV (1.00 < fe/mcIV < 2.00)
 2 - femur about the same length or shorter than
 metacarpal IV (fe/mcIV < 1.00)

1. Metatarsal III, proportional length relative to tibia length (Kellner 2001: character 64):

0 - more than 30% of tibia length

1 - less than 30% of tibia length

1. Pedal digit V, number of phalanges (Kellner 2001: character 65):

0 - with four phalanges
 1 - with 2 phalanges
 2 - with 1 or no phalanx (extremely reduced)

1. Pes, second phalanx of digit V, shape (modified from Kellner 2001: character 66):

0 - reduced or absent
 1 - elongated, straight
 2 - elongated, curved
 3 - elongated, very curved (boomerang shape)
